# Correction: Lower Vocal Tract Morphologic Adjustments Are Relevant for Voice Timbre in Singing

**DOI:** 10.1371/journal.pone.0138601

**Published:** 2015-09-16

**Authors:** The PLOS ONE Staff

## Notice of Republication

This article was republished on September 1, 2015, to correct errors that occurred during the final formatting of the manuscript. The authors apologize for these formatting errors. Please download this article again to view the correct version. The originally published, uncorrected article and the republished, corrected article are provided here for reference.

## Supporting Information

S1 File Originally published, uncorrected article. (PDF)

S2 File Republished, corrected article. (PDF)
