# Supplementary material for: Lower Vocal Tract Morphologic Adjustments Are Relevant for Voice Timbre in Singing
Source: PLoS One. 2015 Jul 17;10(7):e0132241. doi: 10.1371/journal.pone.0132241 (PMC4505946; doi:10.1371/journal.pone.0132241)
Supplement: S1 Table — Table showing the raw data of the endolaryngeal area (ELA) and volume (ELV), the hypopharyngeal area (HPA) and volume (HPV) and the laryngeal height (LH) in speech-like phonation and singing for all vowels (LH in mm, area measures in mm 2, volume measures in mm 3). (PDF) [file pone.0132241.s001.pdf]

Subject No.	1	2	3	4	5	6	7	8	9	10	11	12	13
**ΔHI (dB)**	**-4,1**	**6,9**	**2,1**	**6,3**	**5,0**	**4,7**	**2,9**	**17,6**	**3,7**	**-0,1**	**13,5**	**2,9**	**-2,5**
ELA /a/ speech	98,7	90,0	75,4	95,7	143,9	113,9	103,5	93,8	59,0	65,6	128,6	52,7	101,5
ELA /e/ speech	138,0	90,1	157,8	110,9	172,3	109,2	98,2	88,8	59,6	69,3	110,6	56,2	98,2
ELA /i/ speech	136,9	114,8	91,8	86,7	169,0	101,3	118,5	116,0	52,3	76,1	119,4	83,1	115,4
ELA /o/ speech	182,3	129,8	120,2	123,8	164,4	130,0	126,1	109,0	88,1	103,3	142,1	101,1	91,2
ELA /u/ speech	208,2	110,3	163,8	197,6	170,4	113,6	122,1	124,1	80,0	100,6	135,0	121,3	119,1
ELA /a/ singing	93,8	166,8	89,6	157,7	108,7	111,1	108,8	69,6	55,1	57,9	172,2	60,3	139,3
ELA /e/ singing	100,0	140,1	111,9	184,3	206,6	93,8	99,0	64,4	62,1	75,2	174,7	74,9	101,3
ELA /i/ singing	123,9	139,9	139,3	185,8	187,8	78,7	96,2	81,6	48,7	76,5	190,5	104,7	161,6
ELA /o/ singing	92,0	151,3	115,4	177,3	172,0	131,8	148,3	123,2	57,4	103,3	159,3	123,0	147,0
ELA /u/ singing	142,3	160,0	108,9	128,7	200,6	129,1	156,8	136,6	62,1	110,4	183,6	120,3	209,2
ELV /a/ speech	1294	889	567	1253	1234	1267	1293	1106	843	762	970	765	1051
ELV /e/ speech	1549	714	1133	1068	1561	1378	1058	894	867	841	859	783	709
ELV /i/ speech	1479	1088	665	1093	1191	1001	1226	1250	847	1094	951	1132	1037
ELV /o/ speech	1920	816	640	1055	1365	1183	1233	1241	1067	1165	945	1234	1048
ELV /u/ speech	2288	1003	1170	1897	1185	1159	951	1275	1163	1153	867	1228	1139
ELV /a/ singing	1469	1191	497	1395	1263	1251	1037	811	797	634	990	987	951
ELV /e/ singing	1235	1198	888	1900	2024	980	1019	870	770	830	1004	985	882
ELV /i/ singing	1355	1330	1114	1566	1324	841	1052	870	598	952	1541	1296	1291
ELV /o/ singing	1151	993	721	1353	1540	1116	1350	1434	940	940	1126	1084	1356
ELV /u/ singing	1873	1313	732	1007	1554	1067	1115	1205	864	1348	1438	1512	2042
HPA /a/ speech	326,4	275,1	161,5	234,4	320,6	408,1	326,7	196,9	157,6	192,4	502,9	257,6	279,6
HPA /e/ speech	453,5	363,3	562,3	312,5	583,7	472,2	411,8	289,3	249,3	307,1	565,3	340,9	473,0
HPA /i/ speech	488,1	418,8	571,0	316,4	541,9	475,8	475,8	309,1	225,0	318,3	685,7	465,9	464,7
HPA /o/ speech	495,5	555,1	305,9	295,6	524,9	487,6	401,8	318,3	277,2	332,4	539,8	468,1	443,6
HPA /u/ speech	678,3	419,0	520,9	472,5	489,8	486,7	497,1	345,0	312,2	345,1	576,8	613,1	517,3
HPA /a/ singing	399,6	531,7	230,6	305,5	387,7	455,1	381,3	248,8	199,8	234,2	634,7	247,8	445,0
HPA /e/ singing	432,5	588,7	578,7	495,4	639,1	466,8	425,1	318,4	331,7	341,1	796,2	388,7	523,4
HPA /i/ singing	501,4	548,4	652,2	479,2	796,9	497,8	489,6	418,0	299,2	376,4	860,0	513,8	618,0
HPA /o/ singing	391,1	573,1	414,7	427,4	594,1	492,4	519,0	429,9	242,8	406,9	794,6	410,7	592,5
HPA /u/ singing	500,9	538,3	603,0	321,8	760,8	579,3	495,3	569,6	341,4	441,2	858,5	621,7	702,9
HPV /a/ speech	4003	2959	3007	2940	3787	4715	3551	2583	1927	2342	5032	2902	3629
HPV /e/ speech	5138	3866	5722	3314	5560	5231	3922	3086	2272	3132	5405	3259	4605
HPV /i/ speech	5510	4586	5908	3286	6012	5029	4439	3173	2299	3451	6261	4477	4616
HPV /o/ speech	5787	5532	4391	3198	5265	5403	3986	3628	3044	3743	5367	4648	4333
HPV /u/ speech	7498	4475	5678	4850	5633	5390	4700	3808	3350	3652	5625	6087	4813
HPV /a/ singing	4389	5197	3365	3741	4103	4787	3876	2900	2310	2555	6177	2889	4644
HPV /e/ singing	4519	5916	5719	4841	6193	5141	3933	3270	2902	3361	7181	3941	5185
HPV /i/ singing	5443	5663	6732	4832	6773	5262	4639	3926	2759	3587	8345	4953	6198
HPV /o/ singing	4165	5692	5174	4571	7835	5110	5500	4318	2924	4235	6593	4657	5761
HPV /u/ singing	5854	5672	6304	3442	8297	5854	5084	5522	3462	4526	8203	6172	7100
LH /a/ speech	19	17	39	32	35	21	14	45	21	28	29	16	32,7
LH /e/ speech	26	32	36	33	32	20	24	45	28	25	23	19	35,4
LH /i/ speech	16	30	37	33	29	23	24	46	27	25	27	29	34,6
LH /o/ speech	25	23	38	31	33	17	16	43	20	20	28	20	27,8
LH /u/ speech	16	28	34	20	34	15	18	43	24	22	27	16	28,5
LH /a/ singing	8	38	40	28	22	14	8	39	19	14	6	24	20,4
LH /e/ singing	15	17	38	30	17	18	15	41	23	15	-2	27	23,9
LH /i/ singing	9	26	38	28	19	20	13	38	21	13	-3	28	24,3
LH /o/ singing	15	18	38	20	14	18	0	31	18	6	-1	28	15,0
LH /u/ singing	7	22	29	30	19	7	1	28	16	9	-4	32	17,2
