# Supplementary material for: Correction: Lower Vocal Tract Morphologic Adjustments Are Relevant for Voice Timbre in Singing
Source: PLoS One. 2015 Sep 16;10(9):e0138601. doi: 10.1371/journal.pone.0138601 (PMC4574034; doi:10.1371/journal.pone.0138601)
Supplement: S1 File — (PDF) [file pone.0138601.s001.pdf]

RESEARCH ARTICLE

Lower Vocal Tract Morphologic Adjustments Are Relevant for Voice Timbre in Singing

Alexander Mainka[1,2]*, Anton Poznyakovskiy[3], Ivan Platzek[4], Mario Fleischer[3], Johan Sundberg[5], Dirk Mürbe[1,2]

1 Division of Phoniatrics and Audiology, Department of Otorhinolaryngology, University Hospital Carl Gustav Carus, Technische Universität Dresden, Dresden, Germany, **2** Voice Research Laboratory, Hochschule für Musik Carl Maria von Weber, Dresden, Germany, **3** Department of Otorhinolaryngology, University Hospital Carl Gustav Carus, Technische Universität Dresden, Dresden, Germany, **4** Department of Radiology, University Hospital Carl Gustav Carus, Technische Universität Dresden, Dresden, Germany, **5** Department of Speech, Music and Hearing, School of Computer Science and Communication, KTH CSC, Stockholm, Sweden

* alexander.mainka@uniklinikum-dresden.de

CrossMark
click for updates

OPEN ACCESS

Citation: Mainka A, Poznyakovskiy A, Platzek I, Fleischer M, Sundberg J, Mürbe D (2015) Lower Vocal Tract Morphologic Adjustments Are Relevant for Voice Timbre in Singing. PLoS ONE 10(7): e0132241. doi:10.1371/journal.pone.0132241

Editor: Johan J Bolhuis, Utrecht University, NETHERLANDS

Received: December 18, 2014

Accepted: June 12, 2015

Published: July 17, 2015

Copyright: © 2015 Mainka et al. This is an open access article distributed under the terms of the Creative Commons Attribution License, which permits unrestricted use, distribution, and reproduction in any medium, provided the original author and source are credited.

Data Availability Statement: The analyzed data are available at: http://dx.doi.org/10.5061/dryad.r22h4.

Funding: We acknowledge support by the German Research Foundation and the Open Access Publication Funds of the TU Dresden. The funders had no role in study design, data collection and analysis, decision to publish, or preparation of the manuscript.

Competing Interests: The authors have declared that no competing interests exist.

Abstract

The vocal tract shape is crucial to voice production. Its lower part seems particularly relevant for voice timbre. This study analyzes the detailed morphology of parts of the epilaryngeal tube and the hypopharynx for the sustained German vowels /a/, /e/, /i/, /o/, and /u/ by thirteen male singer subjects who were at the beginning of their academic singing studies. Analysis was based on two different phonatory conditions: a natural, speech-like phonation and a singing phonation, like in classical singing. 3D models of the vocal tract were derived from magnetic resonance imaging and compared with long-term average spectrum analysis of audio recordings from the same subjects. Comparison of singing to the speech-like phonation, which served as reference, showed significant adjustments of the lower vocal tract: an average lowering of the larynx by 8 mm and an increase of the hypopharyngeal cross-sectional area (+ 21:9%) and volume (+ 16:8%). Changes in the analyzed epilaryngeal portion of the vocal tract were not significant. Consequently, lower larynx-to-hypopharynx area and volume ratios were found in singing compared to the speech-like phonation. All evaluated measures of the lower vocal tract varied significantly with vowel quality. Acoustically, an increase of high frequency energy in singing correlated with a wider hypopharyngeal area. The findings offer an explanation how classical male singers might succeed in producing a voice timbre with increased high frequency energy, creating a singer`s formant cluster.

Introduction

Voice production involves the generation of a pulsating transglottal airflow which is filtered by the vocal tract (VT) resonator. The shape of the vocal tract defines formant frequencies and the frequency response of the filter which, in turn, defines vowels, consonants and essential parts of voice timbre [1]. Timbre in the following is used in the sense described by Helmholtz for whom differences of overtone intensitiy were decisive for the percieved tone colour [2]. Plomp

later perceived timbre of steady-state sounds as determined by the frequency spectrum [3]. Dynamic aspects of sound production are left out in these definitions and are also not considered in the present study which will be concentrating on sustained vowels. There are important differences between the timbre characteristics of spoken and sung vowels. In speech science the term voice quality or timbre is commonly understood as a characteristic perceived color of an individual voice. In contrast in classical singing style the singer`s formant cluster is a prominent timbre component of male voices. Acoustically, it is a a clustering of energy in the frequency range of 3 kHz, first observed by Bartholomew [4].

Is has been shown that the lower part of the VT is important for voice timbre particularly for the creation of the singers formant cluster [5]. More specifically, the epilaryngeal tube and the hypopharyngeal area should be decisive. Acoustically the epilaryngeal tube can be regarded as a Helmholtz-resonator, which ends in the pharynx. Its resonance frequency will be almost entirely determined by the epilaryngeal tube if the area ratio between the outlet and the pharynx at the level of the outlet will be smaller than 1:6 [6]. In two independent model experiments, the resonance frequency of the epilaryngeal tube has been found to lie in the vicinity of 2.8 kHz [5,7]. This would place it between the frequencies of formants 3 (2.2–3.0 kHz) and 4 (3.2–3.5 kHz) in adult male speech [5], thus creating a formant cluster that boosts the spectrum level in this frequency region. A small area ratio between the laryngeal outlet and the pharynx should keep the larynx tube resonance independent of the remaining part of the VT. Greater area ratios will have the consequence of making the larynx tube resonance more sensitive to the rest of the VT.

Many of the theories of the acoustic and morphometric principles of vocal tract configuration in singing are based on simplified physical or numerical models [5,8]. Hence, the morphological details of the VT in singing are not known due to the lack of accurate three-dimensional measurements. In contrast to former imaging facilities, current magnetic resonance imaging (MRI) allows the acquisition of high-resolution images of the VT without radiation exposure. This possibility has been used extensively, mainly for speech [9,10], but also for singing [11].

A detailed analysis of VT shape requires 3D imagery and stable articulatory conditions, i.e. the analysis of sustained vowels as has been used in some previous studies [12–16]. The only volumetric study comparing different sustained vowels showed no significant changes in the endolaryngeal and the lower hypopharyngeal region from one vowel to another [7].

The present study addresses two main questions: Is there a systematic adjustment of the lower VT morphology in singing as compared to speech-like phonation? Is there a systematic displacement of the larynx comparing singing and speech-like phonation? More specifically, cross-sectional area and volume measures of the epilaryngeal and hypopharyngeal regions were analyzed, which are commonly assumed to be particularly important to voice timbre in singing.

Materials and Methods

Ethics statement

The study was approved by the Ethics Committee of the Faculty of Medicine of the Technische Universit ät Dresden (No. 402.11). All participants gave their written consent.

Subjects and data aquisition

Thirteen male singing students participated as subjects. Information about their age, voice category and previous singing experience are given in Table 1. All of them were at the beginning of their professional solo singer education at the Hochschule für Musik Carl Maria von Weber, Dresden, Germany. They were asked to produce five sustained German vowels, /a/, /e/, /i/, /o/ and

Table 1. Age, years (yr) of prior singing lessons, voice category and values of the difference of the Hammarberg Index in speech and singing (HI) for all subjects including mean values.

Subject No.	Age (yr)	Singing lessons (yr)	Voice category	HI (dB)
1	20	10	baritone	-4.1
2	20	4	baritone	+6.9
3	21	3	tenor	+2.1
4	21	6	tenor	+6.3
5	21	10	baritone	+5.0
6	19	1	baritone	+4.7
7	20	4	baritone	+2.9
8	19	10	baritone	+17.6
9	21	10	tenor	+3.7
10	19	10	tenor	-0.1
11	20	1	baritone	+13.5
12	20	3	tenor	+2.9
13	20	2	baritone	-2.5
mean	20.1	5.7	-	+4.5

doi:10.1371/journal.pone.0132241.t001

/u/ at the pitch of A 3 (220 Hz), with a medium loudness, in two different phonatory conditions: first, in a natural, speech-like phonation mode and second, in a resonant, projected phonation mode, as in singing in the classical style. They were also asked to reduce their vibrato without abandoning the classical singing style. The pitch reference was provided and checked by means of a pitch pipe. Both tasks were produced sequentially in a 3 T MRI machine (Magnetom Trio Tim, Siemens Medical Solutions, Erlangen, Germany). The MRI was performed with a 12-element-head-neck-coil using a 3D volume-interpolated-breathhold-examination sequence with 1:3 ms/3:8 ms (echo time/repetition time), flip angle 10, a field-of-view of 250 x 250 mm, and a matrix of 192 x 192. The MRI recording was initiated as soon as the subject had started phonation. For each vowel sequence, the acquisition time was restricted to 9:2 s. The experiment yielded ten recordings per subject, five vowels with two takes each, all produced in supine position. The singers were instructed to keep articulation constant during each vowel and to inform the examiner if they felt that they failed to do so. In latter cases, the recording was repeated. For each vowel sequence, a set of 52 sagittal slices of the whole VT was obtained. The slice thickness was 1:5 mm. The obtained resolution of the images was 1:3 mm. Due to the known limitations of the MRI recording technique, the teeth were not detected. Since the target of the study was adjustments of the lower VT, the influence of the teeth on the acoustics of the interesting frequency range of about 3 kHz also seemed to be negligible.

Immediately after the MRI data acquisition, audio recordings of the same tasks were made in an adjacent room, under matching conditions. As in the MRI session, the subjects were recorded in a supine position. The audio signal was recorded by an electric condenser microphone (EM900 from Tbone, Germany), which has a relatively flat frequency response in the interesting range from 0:2 10 kHz. The microphone was placed at a distance of 30 cm from the mouth and recordings were aquired to a computer with Audacity software [17] at a sampling rate of 44:1 kHz/16 bit.

Processing, segmentation and acoustical analysis of MRI data

To facilitate the segmentation, the images were stacked and scaled along all three axes by a factor of four with ImageJ (National Institutes of Health, Bethesda/MD, USA) using bilinear interpolation and resulting in 208 images with 768x768 pixel each. Then the images were processed

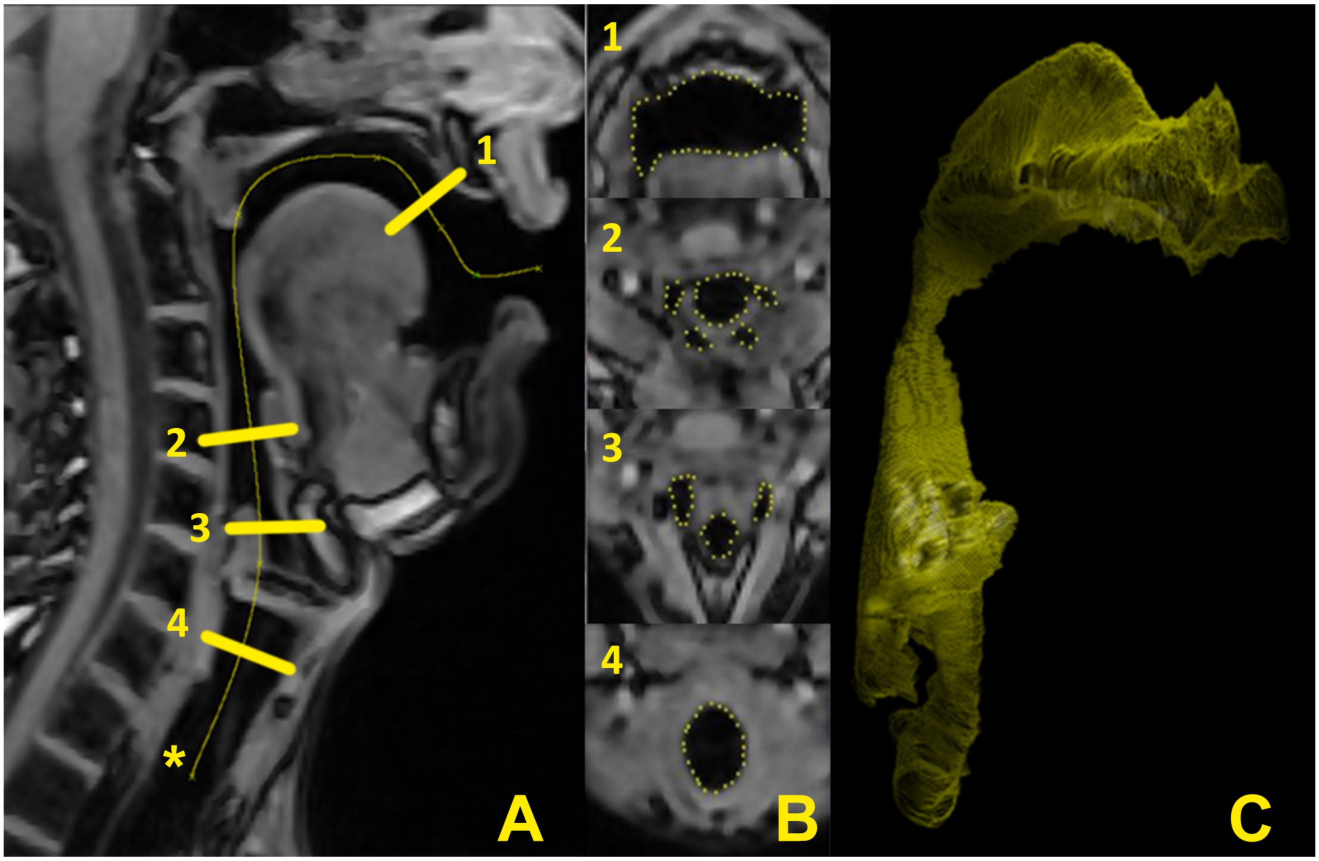

Fig 1. From the MRI to the segmented vocal tract. Typical example of the centerline (*) position used for segmentation (A). Four examples of cross-sections at different levels, as indicated (B). The corresponding 3D representation of the segmented vocal tract (C).

doi:10.1371/journal.pone.0132241.g001

with anisotropic diffusion by means of IPTools [18]. A centerline was defined in the mid-sagittal plane. In order to obtain a well defined and easily reproduceable image stack of the lower VT, the centerline was defined in a standardized manner by using two anatomical landmarks: the tip of the uvula and the crossing of the arytenoids with the ventricular folds (see Fig 1). The image stack was resampled with a fixed distance of 1 pixel between images to a set of up to 780 images, whose planes were normal to the centerline. The distance from one segment to the next was 0:33 mm, i.e., 30 segments represented approx. 10 mm. The resulting images were segmented along the air-tissue-border by help of active contours in a semiautomatic procedure [19,20]. To ensure a realistic representation of the VT anatomy, the resulting segments were checked and if needed modified manually on a single-point basis by an experienced laryngologist. The resulting two-dimensional segmentations, which correspond to the area function of the stacked VT, allowed for a detailed analysis of 2D and 3D measures within the centerline-based coordinate system.

Definition of morphologic parameters

Two cross-sectional areas and two volume measures were selected. The area measurements were derived from single cross sections taken directly from the centerline-based segmentation (see Fig 2).

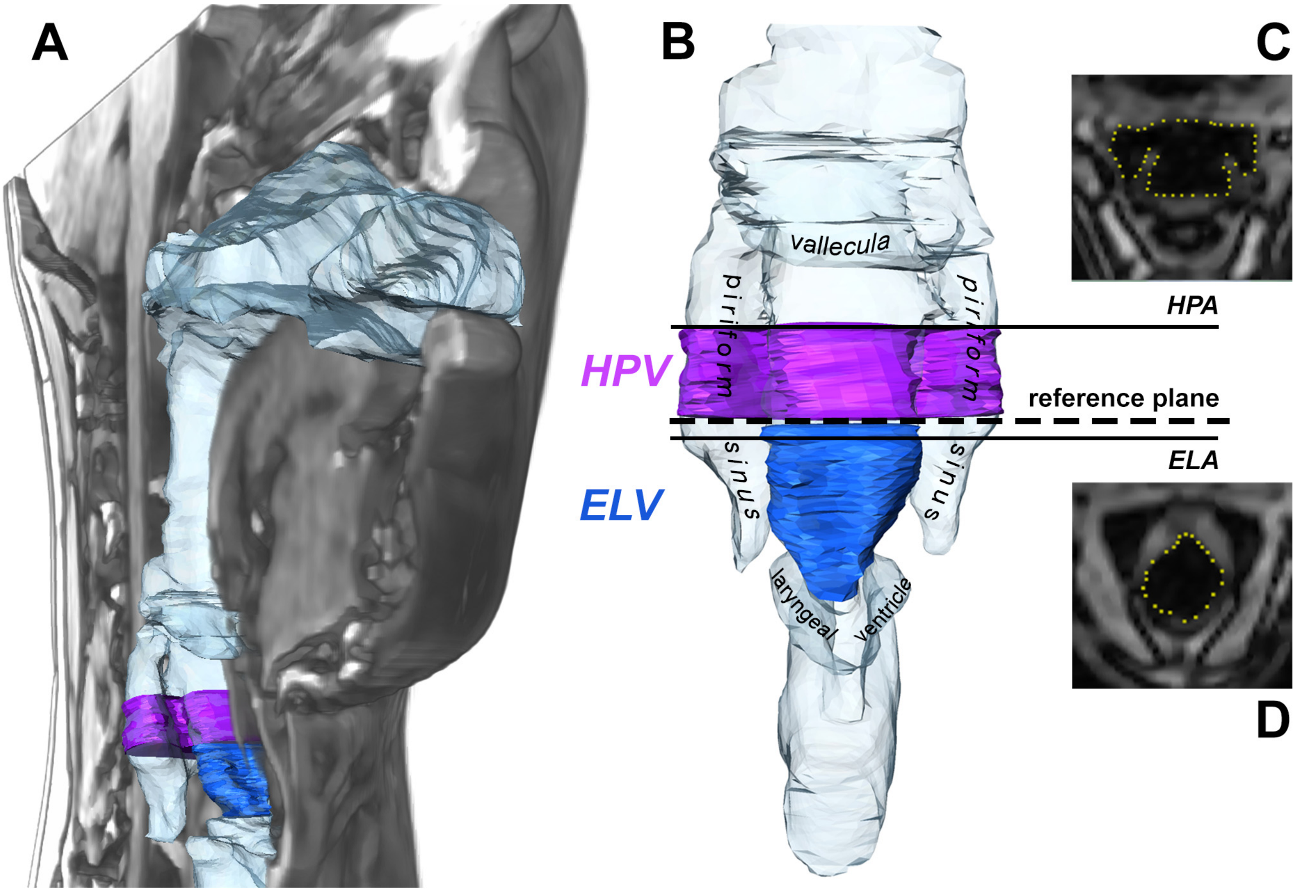

Fig 2. Visualization of the analyzed vocal tract measures. (A) 3D model of the complete vocal tract in a right-oblique projection within a midsagital section of the corresponding MRI data. (B) 3D model of the lower vocal tract showing the location of the reference plane at the top of the arytenoids, the selected volume measures: in purple: hypopharyngeal volume (HPV = volume from reference plane 10 mm upwards), in blue: endolaryngeal volume (ELV = volume from reference plane to lower ventricular folds) as well as the position of the area measures: hypopharyngeal area (HPA) and endolaryngeal area (ELA) with the corresponding segmented MR images (HPA/(C) and ELA/(D)).

doi:10.1371/journal.pone.0132241.g002

A reference plane, representing the exit of the epilarynx, was identified as the first segment just below the uppermost complete posterior closure of the arytenoid cartilages. An endolaryngeal cross-sectional area (ELA) was measured five slices (1:7 mm) below this plane within the epilarynx tube. The second area measure reflecting the hypopharyngeal width was taken 30 slices (10 mm) above the reference plane. This area is mostly located slightly below the valleculae and the top part of the piriform sinuses. It typically showed the hypopharyngeal width at the level of the top part of the piriform sinuses. Henceforth it will be referred to as the hypopharyngeal area (HPA).

Two corresponding volume measures were defined, one epilaryngeal and one hypopharyngeal. The inferior limit of the former was located at the level of the ventricular folds, where the distance between them reached its minimum, while the epilarynx exit reference plane was chosen as its upper limit. Henceforth this volume measure will be called the endolaryngeal volume (ELV). As a consequence of the dynamic definition of the lower border of the ELV, the resulting height of this volume varied between 6 and 14:5 mm. The hypopharyngeal volume (HPV) was measured between the HPA and the epilarynx exit reference plane thus representing the portion of the lower pharynx just above the larynx including the upper piriform sinuses. For the area and volume measures, the percental change between the two phonation modes was

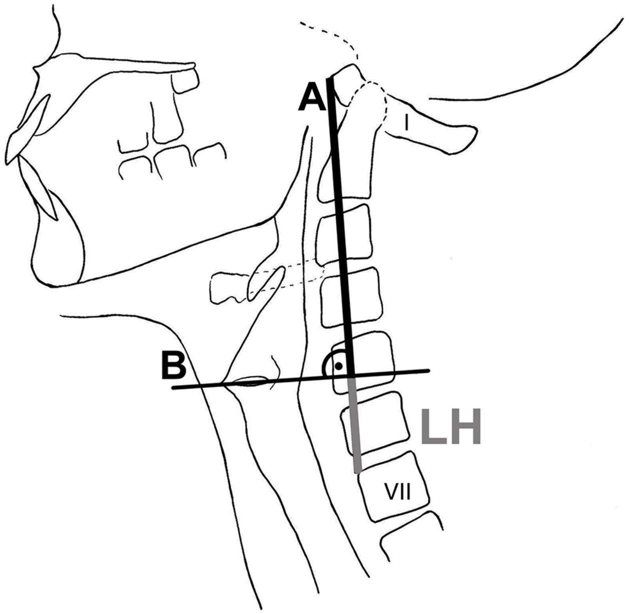

Fig 3. Schematic illustration of helplines A and B. Projection of the helplines A and B on the cervical spine and larynx structures. The helplines served for measuring the LH. `I' and `VII' denote the respective vertebra.

doi:10.1371/journal.pone.0132241.g003

calculated as follows:

$$\frac{measure_{singing}}{measure_{speech-like}} \times 100 = measure_{pc} \ (\text{e.g.HPA}_{\text{pc}}).$$

That means, relative measures for singing were determined by utilizing the natural, speech-like phonation as reference mode. Also, the ratios for the two area measures ELA = HPA and for the two volume measures ELV = HPV were determined for each configuration, i.e., for each combination of phonation mode and vowel.

The position of the larynx was measured in relation to non-mobile parts of the VT. A helpline A was drawn from the anterior rim (tuberculum anterius) of the first cervical vertebra (atlas) to the upper anterior rim (margo anterior superior) of the 7th cervical vertebra. Normal to this line a second line B was drawn that touched the superior edge of the vocal folds at the anterior laryngeal commissure. Laryngeal height (LH) was defined as the distance between the intersection of helpline A and B and the upper anterior rim of the 7th cervical vertebra (see Fig 3).

Analysis of the audio recordings

To obtain quantitative data on the extent to which the singers had developed a singer's formant cluster, long-term-average spectrum analysis (LTAS) (range 0–5:5 kHz, bandwidth 250 Hz) of the audio recording of each vowel sequence was run.

The spectrum slope is heavily influenced by vocal loudness, and most speech-like and singing samples differed substantially in this respect. The effects of such differences on LTAS curves can be approximated by means of an equation, which multiplies the difference in equivalent sound level with a frequency dependent gain factor [21]. To allow for a fair comparison, the LTAS for the speech-like samples were compensated by using this equation.

Then, the Hammarberg index (HI) was determined for speech-like and sung vowels. The index is defined as the level difference between the highest spectrum peak in the 0–2 kHz range and the highest spectrum peak in the 2–5 kHz range [22]. Other authors reused this index as singing power ratio [23]. A prominent spectrum peak above 2 kHz typically observed in male classical singing is thereby represented by a low or even negative HI. The peak can be explained as the acoustical consequence of clustering formants 3, 4, and 5, and is hence referred to as the singer's formant cluster [5]. To describe the relative increase of the singer`s formant cluster the level difference in dB of HI between speech-like and singing was then calculated as $\Delta HI = HI_{speech-like} - HI_{singing}$. This means that high HI values reflected a more prominent singer`s formant cluster in singing compared to speech-like phonation.

Statistics

A two-way repeated measure analyses (ANOVA) was carried out on the following measures: ELA, HPA, ELV, HPV, LH and the quotients ELA = HPA, and ELV = HPV with `phonation mode' (variables: speech-like and singing) and `vowel' (variables: /a/, /e/, /i/, /o/ and /u/) as within-subject factors. To look for correlations between HI and LH and area and volume measures the coefficient of determination (R^2) was applied.

Estimation of the VT transfer function based on hybrid area functions

To test the influence of the lower VT on the acoustical output an examplary area function hybrid combining the lower VT in singing with the upper VT in speech-like phonation and vice versa was used. The material was taken from the two data sets on vowel /a/ of subject 8, who had a high HI. The obtained area function of the lower VT from the glottis to the inferior-most part of the vallecula in singing was attached to the upper remaining part of the VT taken from speech-like. The inferior parts of the piriform sinuses besides the larynx were disregarded, following the method used by Takemoto [7]. Likewise, the upper VT in singing was fused to the lower VT in speech-like condition. The resulting hybrid area functions as well as the two originals in singing and speech-like condition served for the computation of a one-dimensional acoustical analysis. To calculate this transfer function of the VT the freely available phonetics software PRAAT [24] was used with standard parameters for glottal damping and lip radiation.

Results

The analysis of the acoustical parameters showed stronger singer`s formant clusters in singing, resulting in an average 4:5 dB difference of the HI, even though three of the 13 subjects showed negative values (Table 1).

Larynx height

As mentioned above, a lowered larynx is commonly regarded as typical in male classical singing. The difference in larynx height (LH) between the singing and speech-like phonation mode revealed that the subjects had a lower larynx position in singing, as illustrated in Fig 4. Across vowels and subjects the larynx was lowered by about 8 mm in singing on average. This difference was statistically significant ($p <: 01$), although subject 12, one of the tenors, showed a completely opposite behavior raising his larynx, on average by 12 mm. For the different vowels the absolute values of LH were significantly different. Analysis of the interaction of both within-subject factors `phonation mode' and `vowel' showed no significant effect.

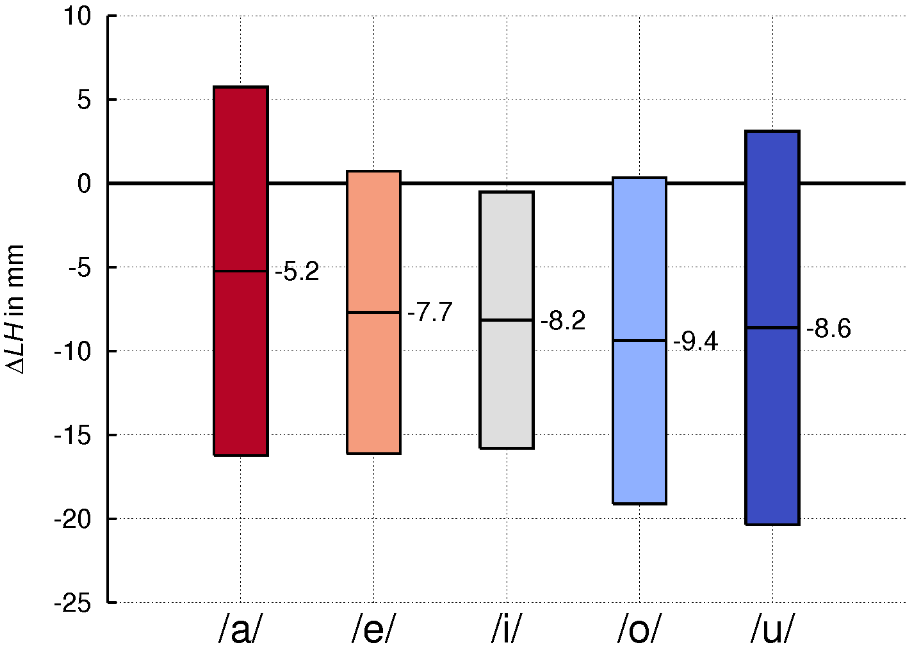

Fig 4. Lowering of larynx position in singing. Values averaged across subjects for the lowering of the larynx (LH for larynx height) in singing compared to speech-like phonation for the indicated vowels. Boxes represent means +/-1SD. Bold base line representing no change in larynx position.

doi:10.1371/journal.pone.0132241.g004

Endolaryngeal area (ELA) and volume (ELV)

Panel A of Fig 5 shows the change in ELA for each vowel, averaged across subjects. For all vowels, the average was greater in singing than in speech-like phonation, the mean difference amounting to + 12:1%. However, this difference failed to reach statistical significance. Panel B of Fig 5 shows the corresponding change of ELV, on average amounting to a 7:2% increase in singing. As expected, the ELV showed a correlation with the ELA, but the correlation varied considerably. The parameters for the linear trendline showed a slope of 9.2 and a coefficient of determination R^2 = 0:3. This variation was due to the dynamic border definition used for calculating the ELV. For both measures vowels differed significantly from each other (p <: 01), whereas there was no significant interaction between `phonation mode' and `vowel'.

Hypopharyngeal area (HPA) and volume (HPV)

A significant increase of HPA (p =: 001) and HPV (p <: 01) was found in singing compared to the reference phonation mode for all vowels, the mean difference amounting to + 21:9% and + 16:8%, respectively (see Fig 6).

A strong correlation between HPA multiplied by the height of HPV (equals to 9:765 mm/ 30 segments) and HPV was found (see Fig 7) with a slope of 0.901 and a coefficient of determination R^2 = 0:92. This suggests that the VT shape in the analyzed hypopharyngeal region shows only small variations regarding its cross sectional area measures and it illustrates the fidelity of the applied segmentation method. For both measures the within-subject factor `vowel' showed a significant effect (p =: 000), whereas there was no significant interaction between `phonation mode' and `vowel'.

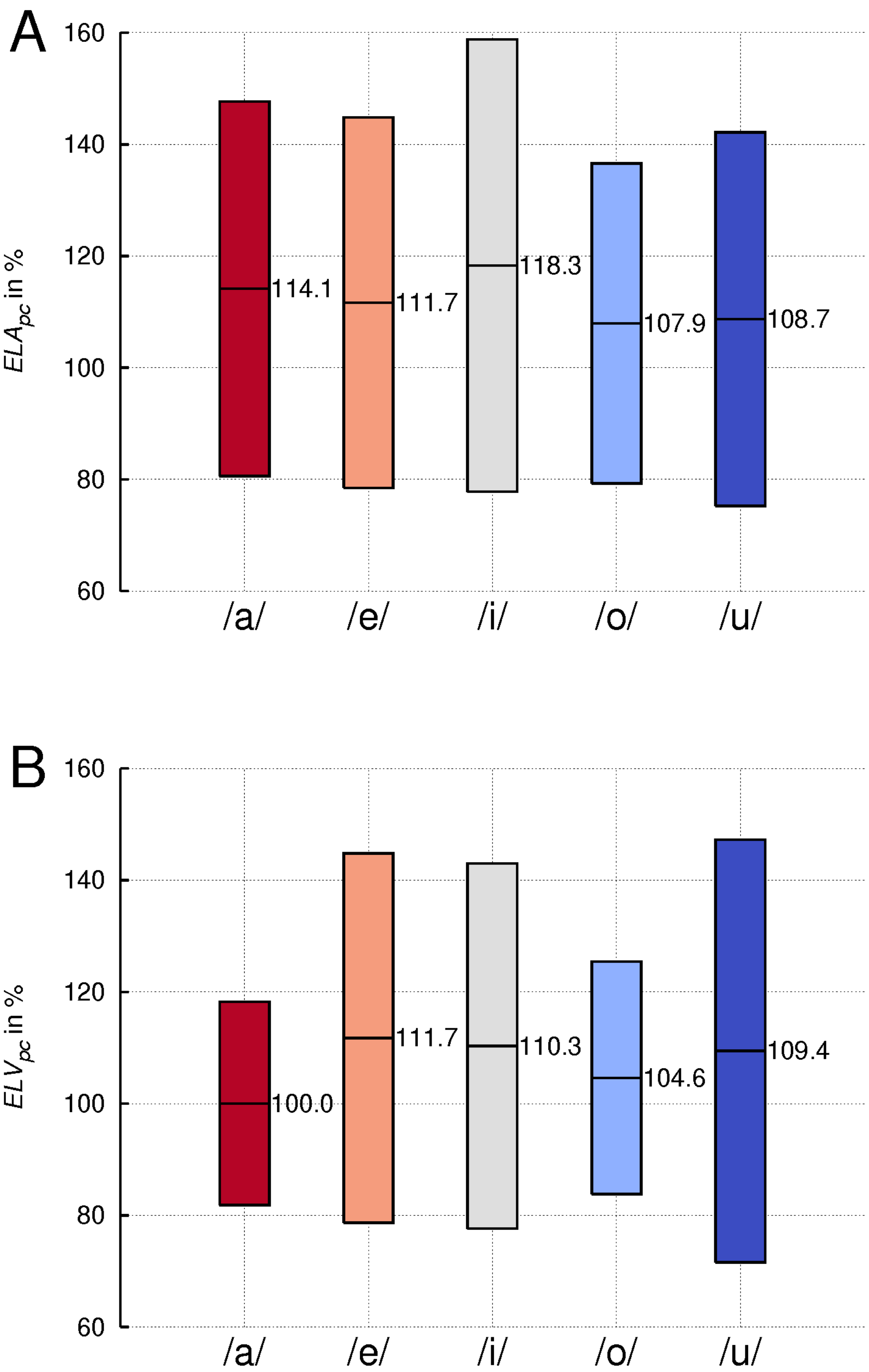

Fig 5. Widening of larynx area and volume in singing. Percentage change (pc) of endolaryngeal area (ELA)(A) and the endolaryngeal volume (ELV) (B) from speech-like phonation to singing, averaged across subjects for the indicated vowels. Boxes represent means +/-1SD.

doi:10.1371/journal.pone.0132241.g005

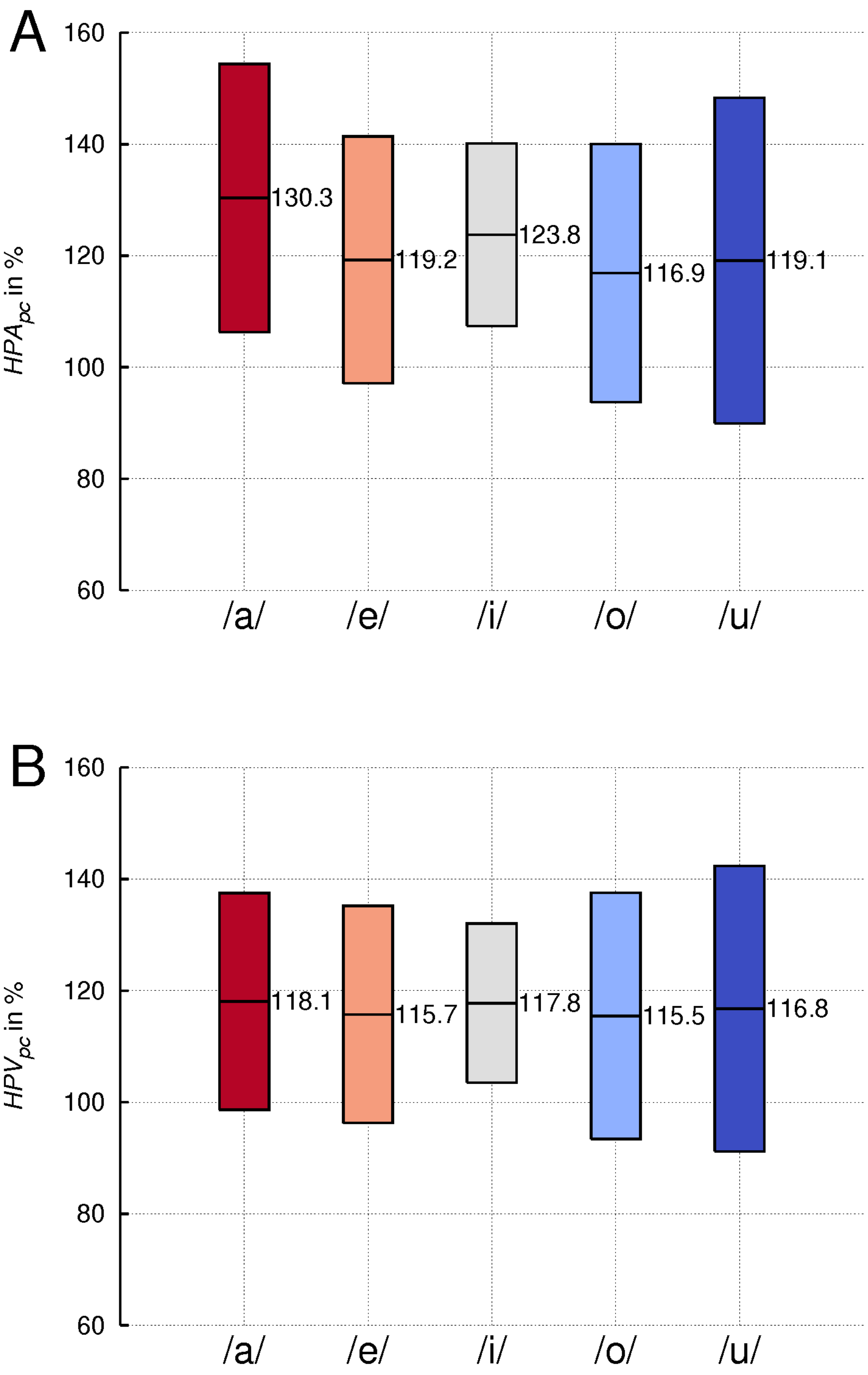

Fig 6. Widening of hypopharynx area and volume in singing. Percentage change (pc) of hypopharyngeal area (HPA)(A) and hypopharyngeal volume (HPV) (B) from speech-like phonation to singing, averaged across subjects for the indicated vowels. Boxes represent means +/-1SD.

doi:10.1371/journal.pone.0132241.g006

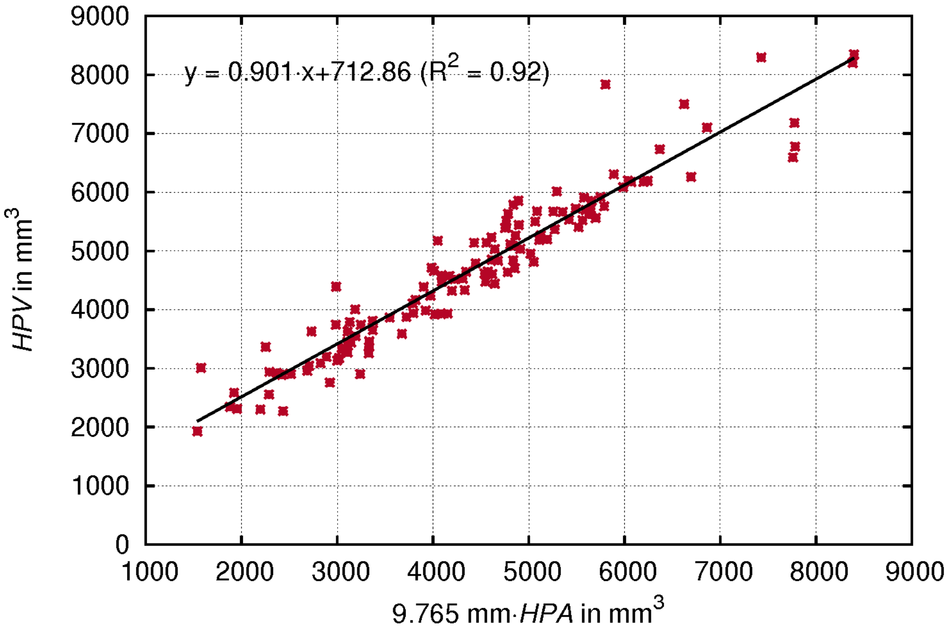

Fig 7. Correlation of hypopharyngeal area and volume measures. Hypopharyngeal area (HPA) multiplied by the height of HPV (9:765 mm) plotted against hypopharyngeal volume (HPV). Individual values for both phonation modes and all vowels.

doi:10.1371/journal.pone.0132241.g007

Larynx-to-pharynx-ratio

As mentioned in the introduction, the larynx-to-pharynx-ratio for the area and volume measures should be acoustically relevant [1, 5]. Even though ELA was greater in singing, the ELA = HPA-ratio was significantly smaller (p =: 05) in singing compared to speech-like phonation (Fig 8A). This was due to an even greater increase of HPA. The mean ratio across subjects and vowels dropped from 0.29 in the speech-like phonation to 0.26 in singing. Again, subject 12 showed a deviant behaviour, increasing the area ratio from 0.19 in speech-like to 0.23 in singing. This was the result of a rather constant HPA and a wider ELA in singing.

Also, the ratio between the two volume measures ELV and HPV showed significantly lower values (p =: 01) in singing compared to speech-like (Fig 8B). The mean values across subjects and vowels were 0.27 in the speech-like phonation and 0.24 in singing. The area and the volume ratio varied significantly with `vowel', yet again the interaction between `voice condition' and `vowel' was not significant.

Correlation of laryngeal height and area and volume measures of the lower VT

Across vowels, no significant correlation between laryngeal height and percentage change of HPA, HPV, ELA and ELV was found. However, for HPA for the vowels /o/ and /u/, which showed the greatest lowering in LH, a clear negative correlation was found: greater lowering of the larynx being associated with a wider pharynx area in singing (see Fig 9). R^2 was -0.30 for the vowel /o/ and -0.36 for the vowel /u/. Both correlations were significant (p <: 05).

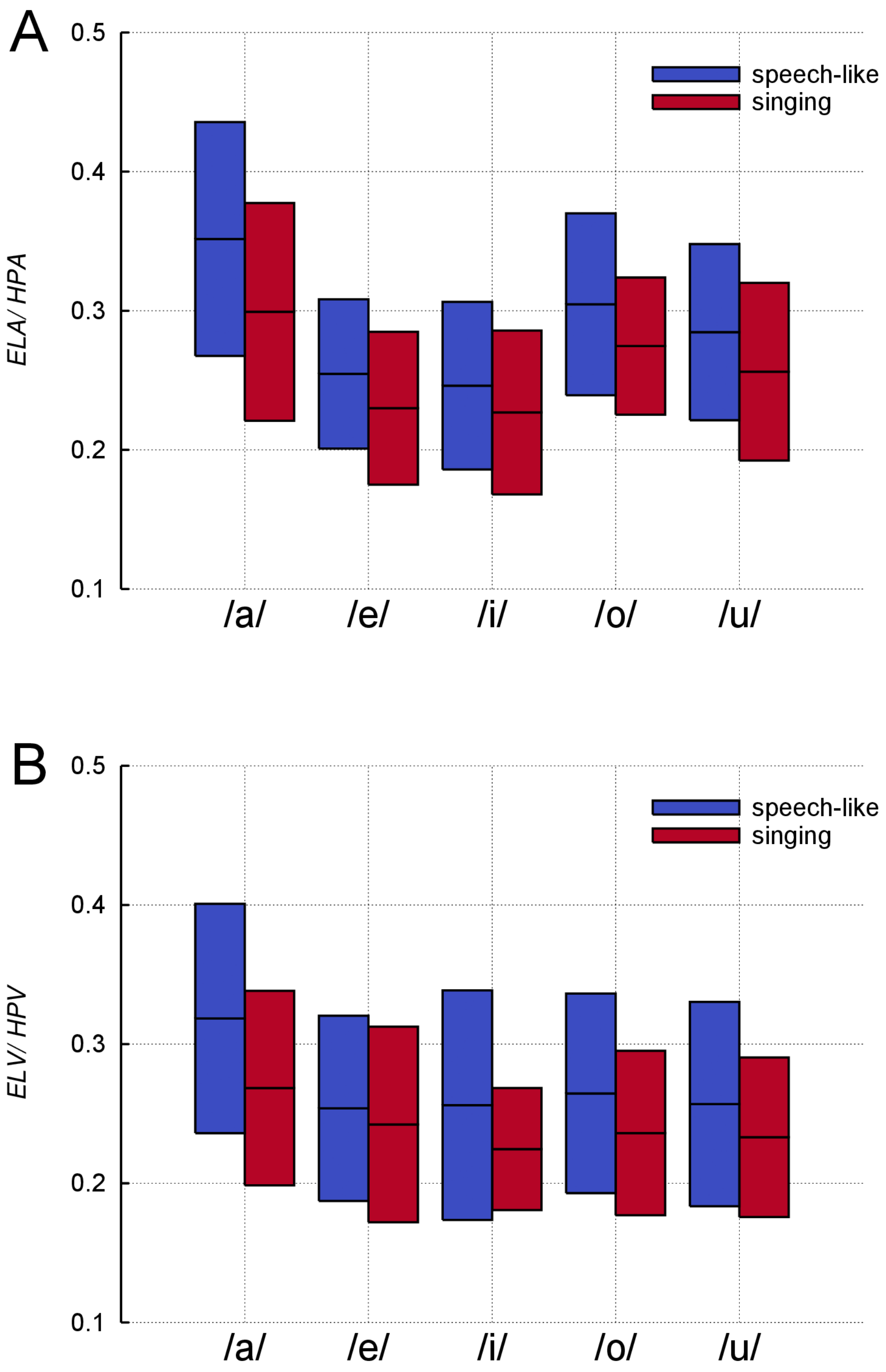

Fig 8. Drop of area and volume ratio in singing. Ratios of the endolaryngeal area (ELA) to the hypopharyngeal area (HPA)(A) and the endolaryngeal volume (ELV) to the hypopharyngeal volume (HPV) (B) averaged across subjects for indicated vowels in speech-like phonation and singing. Boxes represent means +/-1SD.

doi:10.1371/journal.pone.0132241.g008

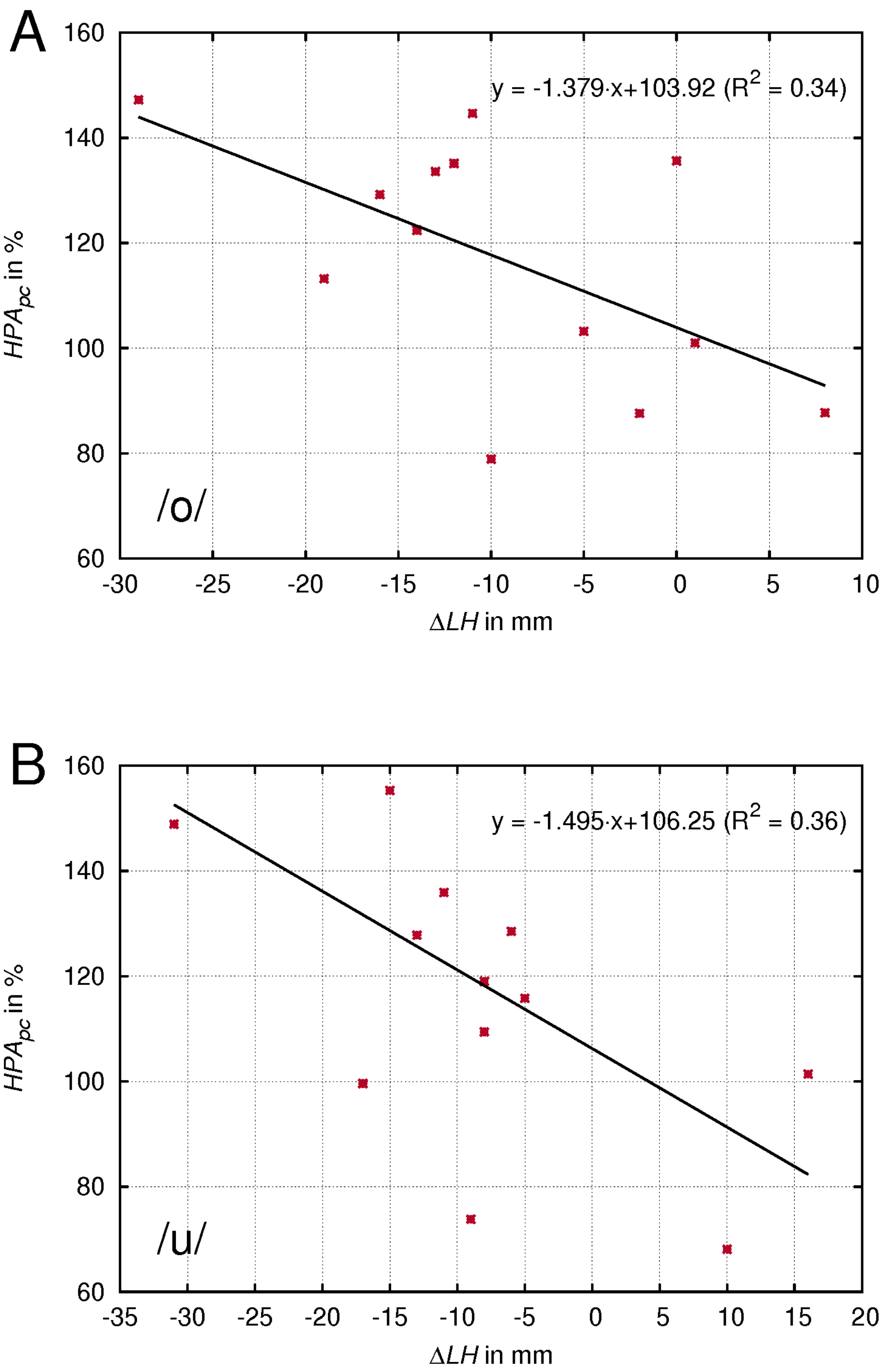

Fig 9. Correlation of the laryngeal lowering to the hypopharyngeal widening. Individual values of the laryngeal lowering (LH) plotted against the percentage change (pc) of the hypopharyngeal area (HPA) in singing compared to the speech-like phonation for the indicated vowels.

doi:10.1371/journal.pone.0132241.g009

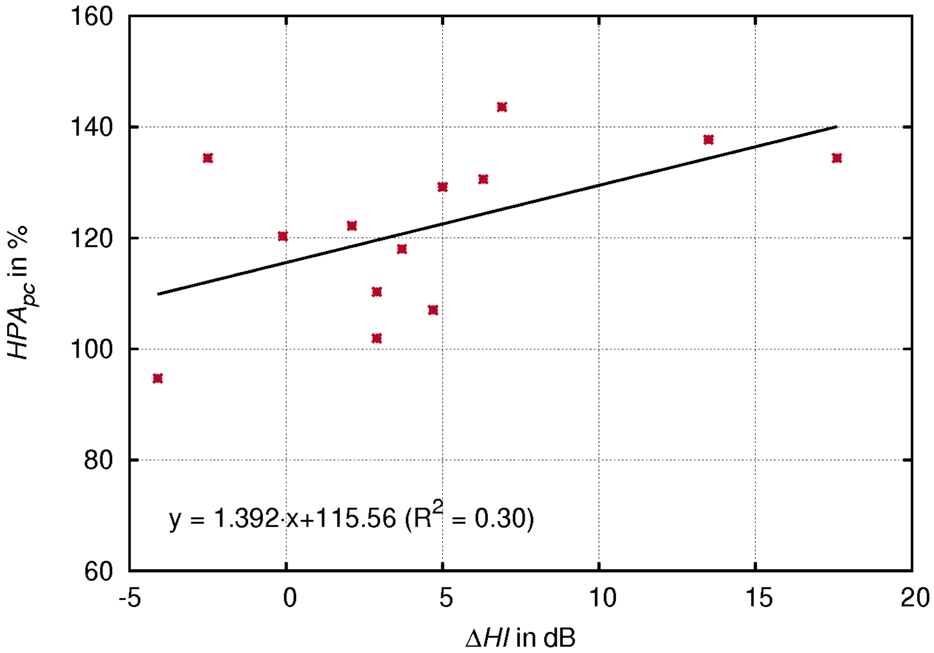

Fig 10. Correlation of acoustics to hypopharyngeal widening. Mean values across all vowels for each subject of percentage change (pc) of the hypopharyngeal area (HPA) and the difference of the Hammarberg Index in speech-like phonation and singing (HI).

doi:10.1371/journal.pone.0132241.g010

Correlation between the change of Hammarberg index and area and volume measures of the lower VT

Subjects who showed a larger increase of HPA in singing tended to have greater change of the Hammarberg index (HI) corresponding to a higher overtone energy in singing than in speech-like phonation (see Fig 10). The values failed to reach statistical significance ($R^2 = 0:3/p < 0:053$). A similar trend was observed for HPV and HI ($R^2 = 0:23/p = 0:1$). Whereas no such correlation was found for the corresponding laryngeal area and volume measures. Also there was no clear correlation between HI and the calculated area or volume ratios.

Example of transfer function based on hybrid area functions

On the basis of the present results, it seemed reasonable to assume a relationship between the higher formants and the area and volume ratios in the lower VT. To further test this assumption, an examplary hybrid VT area function was constructed of subject 8, who had a high HI (see Section 'Materials and Methods'). Comparison of the transfer function based on a hybrid area function—combining the lower VT in singing with the upper VT in speech-like—to the transfer function based on the original VT area function in speech-like phonation showed a lowering of the 4th formant as the main effect (see Fig 11). These results indicate that the lower VT mostly influences the frequency of the fourth formant.

Discussion

The focus of the present study was to examine VT configuration differences in singing as compared to a natural, speech-like phonation, which served as the reference phonation mode. Of special interest were the epilaryngeal tube and the hypopharyngeal area, two regions likely to

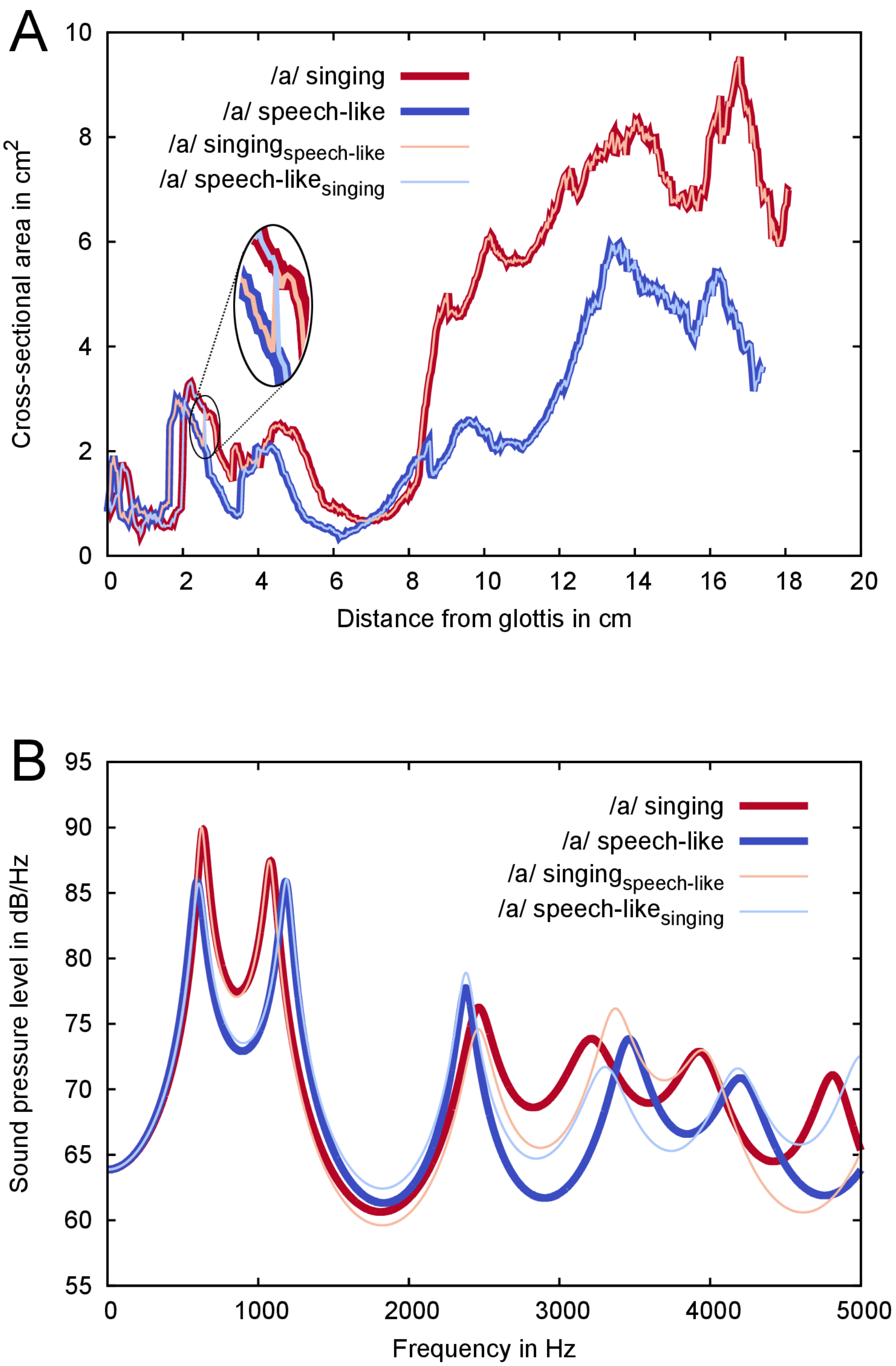

Fig 11. Influence of lower vocal tract (VT) on transfer function. (A) Area functions of the vowel /a/ of subject 8 in the singing (dark red) and the speech-like configuration (dark blue) and the crosswise exchange of the lower VT (slim curves: hybrid of upper VT during singing and lower VT during speech-like phonation in light red and hybrid of upper VT/speech-like and lower VT/singing in light blue). Magnification showing the exchange spot. (B) Transfer functions computed with PRAAT based on the area functions from (A). Colors correspond to the colors used in panel (A).

doi:10.1371/journal.pone.0132241.g011

be particularly relevant to voice timbre [5]. Special care was taken to select clearly defined and easily reproducible measures so as to allow meaningful inter-subject comparisons.

The study is based on data collected by magnetic resonance imaging. 52 images were taken in 9:2 s resulting in a framerate of 5.6 per second. The accuracy of the data is dependent on the MRI tomograph used, the image resolution of the applied sequence including the slice thickness and the stability of the object over time. The influence of the body position in the MRI scanner on the production of sustained vowels was analysed in several studies. Morphologic differences of the VT shape were found for vocally untrained subjects [25,26]. However, trained subjects, as in our study, seemed to be influenced to a lesser extend by postural changes [27]. Influential might have been systematic differences in loudness especially of the singing tasks. Yet with the applied study design vocal loudness could not be monitored. Even though the subjects were instructed to sing with small vibrato, most of them did not avoid it entirely. The vibrato rate is usually approximately 6 Hz. The sampling rate was therefore too small to resolve the vibratory displace-ment of the structures. Another factor might have been movement artefacts during the MRI measurements.

An error estimation was conducted to evaluate the reliability of the morphological VT measures: Considering a computed mean area size of 55 mm^2 as found as minimum in the larynx, assuming a circular area shape (resulting in a diameter of 8:36 mm) and an estimated uncertainty of about 1 mm in diameter possibly caused by vibrato or movement artefacts (resulting in a real diameter of 7:36 mm and 9:36 mm), real area sizes of 42:64 mm^2 and 68:93 mm^2 would result. This corresponds to maximal relative errors of 29% to 20% of the measured cross-sectional area. In case of an area size of 860 mm^2 as found as maximum in the hypopharynx and an uncertainty in diameter of 1 mm, maximal relative errors of 5:8% and 6:3% are obtained. Data supporting the chosen uncertainty value for the lower VT are not available so far. But, it becomes clear that, depending on the absolute size of the measured quantities, the given values for the hypopharynx seem more reliable compared with the values given for the larynx.

Further, for simplification and comparability, only single quantities were taken into account, neglecting detailed 3D morphology. An analysis of the full shape of the interesting structures would probably yield more information about the acoustical relevance but was not considered in this study. In addition, the image processing included a step resulting in a steeper gray scale gradient at the air-tissue-border thus reducing the blurring of contours. Altogether, there are reasons to assume that the accuracy of the MRI-data was sufficient for finding answers to the questions raised.

The Hammarberg Index was selected to quantify the prominence of the singer`s formant in speech-like phonated and sung vowels. A limitation of this index is that it disregards the frequencies of the LTAS peaks. As a consequence, the maximum above 2 kHz might not necessarily represent the singer`s formant cluster. Among the singer subjects, the index differed substantially in both phonation modes. Also, the difference in HI between vowels in speech-like and singing phonation varied greatly, from -4.1 to 17:6 dB. Some of this variation in HI was due to frequency shifts of the most prominent LTAS peaks. This was the case for subject 10, resulting in a negative HI. In the cases of subjects 1 and 13, negative HI values were due to the fact that the main peak below 2 kHz was higher in singing. In reality, the singer's formant peak increased by about 7 dB in singing. In addition, subjects showed different timbre characteristics regarding their speaking voice. Some had a rather high overtone intensity in the singer`s formant frequency region also in their speech-like vowels, thus resulting in a reduced HI although the HI in singing indicated a rather prominent singer`s formant cluster. Likewise, not only the voice quality in singing but also the subjects`speaking manner might have influenced the area and volume differences between the two phonation modes. Well-controlled

experimental conditions regarding vocal style, pitch and vowel quality were applied to reduce that potential source of error. Another presumably influential factor would be the difference in vocal loudness between the two phonation modes, which could not be controlled during the MRI recordings.

Since singers show great inter-individual variability due to e.g. different body measures, voice categories [28] or vocal habits, relative measures were analyzed based on the intra-individual comparison of VT adjustments during vocal tasks rather than absolute measures. This allowed for a meaningful inter-individual comparison including statistical analysis.

On average the larynx was found to be lowered by about 8 mm in singing. In voice pedagogy such lowering is considered essential in the training of classical singing voices [29]. This finding is in qualitative accordance with previously published measurements [30–32].

Configuration changes, particularly of the lower part of the vocal tract, could be expected to underlie the observed acoustical differences between speech-like and sung sustained vowels, as explained in the introduction. The present findings indicate, that the selected laryngeal and hypopharyngeal area and volume measures differed between the two phonation modes. Across subjects and vowels, a significant increase of hypopharyngeal cross-sectional area by 21:9% was found, while the laryngeal area increased by mere 12:1%. Similar observations were made for the respective volume measures resulting in a 16:8% increase of the hypopharyngeal volume and a 7:2% increase of the laryngeal volume in singing. For both laryngeal measures differences in singing were too small to reach statistical significance. Additionally, the laryngeal measures have to be interpreted with caution given the theoretical considerations on measurement errors as outlined above. Still, the present study showed the ratios of laryngeal and hypopharyngeal area measures drop across subjects and vowels from 0.29 in the reference phonation mode to 0.26 in singing. These values are far from the 1 to 6 ratio mentioned in the introduction, but they were getting closer to this value in singing. Correspondingly, the volume ratio was lowered from 0.27 in speech-like phonation to 0.24 in singing. An estimation of the area of the larynx tube opening and the area of the laryngopharynx from MRI images by Detweiler yielded similar area ratios in singing to those found in the present study [31]. The present study was designed to describe dynamic adjustments rather then absolute VT measures. In this respect the measured lowering of the area ratio between larynx and hypopharynx seems to be in accordance with the aforementioned theory in the sense to make better use of the overtone enhancing potential of the inner larynx. Rather unexpected and in contrast to existing literature [7] was the finding that all morphologic measures varied significantly with vowels.

The expansion of the lower vocal tract particularly of the epilarynx tube has been found to influence the characteristics of other types of voice production, for instance in yawning [33]. To sing with a yawny sensation is a commonly used recommendation in voice pedagogy. To create the observed increase of the hypopharynx area, a lowering of the larynx may be a commonly applied strategy. This assumption is supported by the observed correlation between LH and HPA_{pc} for the vowels that showed the greatest lowering of the larynx, /u/ and /o/. Also, there might be other strategies like those which were observed in subject 4 of our study group who combined a raised area ratio in singing with a high HI. Furthermore, there might be other factors contributing to the resulting voice timbre characteristics like mechanical properties of the VT wall [34]. Whether the mechanism is also subject to further refinements of the voice technique during the singer`s education will be investigated by a longitudinal study of the same subjects.

Conclusion

This study investigated differences in the geometry of the lower VT between the two phonatory conditions speech-like and singing by means of high resolution MRI for sustained vowels. The data revealed that the sung vowels as compared with the speech-like vowels were produced with a lower larynx, a greater cross-sectional area and volume of the lower hypopharynx and with a lower ratio of the larynx-to-hypopharynx area and volume. All measures of the lower VT varied significantly with vowel quality. A combination of a lowered larynx and a widened hypopharynx was found in sung versions of the vowels /o/ and /u/. Acoustically, an increase of high frequency energy in singing above 2 kHz correlated with a wider hypopharyngeal area. Additionally an analysis of the VT transfer function with an exemplary hybrid area function showed a down shift of the 4th formant for hybrids with lower VTs in singing configuration. In summary, our findings support the assumption that reducing the acoustic coupling between the larynx tube and the remaining VT is an important component of the articulatory adjustments in male classical singing. Thus by actively widening the hypopharynx singers can enhance the singer`s formant cluster.

Supporting Information

S1 Table. Raw data of the morphologic parameters. Table showing the raw data of the endolaryngeal area (ELA) and volume (ELV), the hypopharyngeal area (HPA) and volume (HPV) and the laryngeal height (LH) in speech-like phonation and singing for all vowels (LH in mm, area measures in mm^2, volume measures in mm^3).
(PDF)

Acknowledgments

The authors are indepted to Hartmut Zabel from the Voice Research Laboratory of the Hochschule für Musik Carl Maria von Weber, Dresden, GERMANY, for collaborating most actively in the recruiting of student singers for this study. We also thank Eberhard Kuhlisch from the Institute for Medical Informatics and Biometry of the Medical Faculty, of the Technische Universität Dresden, GERMANY for performing the statistical tests. Steffi Heinemann and Stephan Schoebel provided much needed assistance during the recordings. Finally, we thank all the participants for their dedication and patience following the given instructions within the MRI.

Author Contributions

Conceived and designed the experiments: AM DM IP. Performed the experiments: AM IP. Analyzed the data: AM JS. Contributed reagents/materials/analysis tools: AM AP MF JS. Wrote the paper: AM JS DM. Designed the software used for the analysis: AP.

References

1. Fant G (1960) Acoustic theory of speech production: Mouton Co., The Hague, The Netherlands.
2. Helmholtz H (1896) Die Lehre von den Tonempfindungen als physiologische Grundlage fr die Theorie der Musik, 5. Auflage, Vieweg und Sohn, Braunschweig.
3. Plomp R. Timbre as a multi-dimensional attribute of complex tones. Frequency Analysis and Periodicity Detection in Hearing; edited by Plomp R. and Smoorenburg G. F. (Sijthoff, Leiden, The Netherlands) 1970.
4. Bartholomew WT (1934) A physical definition of "Good Voice-Quality" in the Male Voice. J Acoust Soc Am 6: 25–33.

5. Sundberg J (1974) Articulatory interpretation of the "singing formant". J Acoust Soc Am 55: 838–844. PMID: 4833080
6. Ingard U (1953) On the Theory and Design of Acoustic Resonator. J Acoust Soc Am 25: 1037–1061.
7. Takemoto H, Adachi S, Kitamura T, Mokhtari P, Honda K (2006) Acoustic roles of the laryngeal cavity in vocal tract resonance. J Acoust Soc Am 120: 2228–2238. PMID: 17069318
8. Titze IR, Story BH (1997) Acoustic interactions of the voice source with the lower vocal tract. J Acoust Soc Am 101: 2234–2243. PMID: 9104025
9. Story BH, Titze IR, Hoffman EA (1996) Vocal tract area functions from magnetic resonance imaging. J Acoust Soc Am 100: 537–554. PMID: 8675847
10. Demolin D, Hassid S, Metens T, Soquet A (2002) Real-time MRI and articulatory coordination in speech. C R Biol 325: 547–556. PMID: 12161933
11. Echternach M, Sundberg J, Arndt S, Markl M, Schumacher M, Richter B (2010) Vocal tract in female registers{a dynamic real-time MRI study. J Voice 24: 133–139. doi: 10.1016/j.jvoice.2008.06.004 PMID: 19185452
12. Takemoto H, Honda K, Masaki S, Shimada Y, Fujimoto I (2006) Measurement of temporal changes in vocal tract area function from 3D cine-MRI data. J Acoust Soc Am 119: 1037–1049. PMID: 16521766
13. Clement P, Hans S, Hartl DM, Maeda S, Vaissiere J, Brasnu D(2007) Vocal tract area function for vowels using three-dimensional magnetic resonance imaging. A preliminary study. J Voice 21: 522–530. PMID: 16581228
14. Echternach M, Sundberg J, Baumann T, Markl M, Richter B (2011) Vocal tract area functions and formant frequencies in opera tenors' modal and falsetto registers. J Acoust Soc Am 129: 3955–3963. doi: 10.1121/1.3589249 PMID: 21682417
15. Echternach M, Popeil L, Traser L, Wienhausen S, Richter B (2014) Vocal tract shapes in different singing functions used in musical theater singing-a pilot study. J Voice 28: 653 e651-653 e657.
16. Kitamura T, Honda K, H. T (2005) Individual variation of the hypopharyngeal cavities and its acoustic effects. Acoust Sci Tech 26: 16–26.
17. Audacity software. Available: http://audacity.sourceforge.net/. Accessed 11 March 2015.
18. IPTools software. Available: freeware,http://www.uniklinikum-dresden.de/das-klinikum/ kliniken-polikliniken-institute/hno/forschung/forschungslabor-gehor/links. Accessed 18 November 2014.
19. Poznyakovskiy AA, Zahnert T, Kalaidzidis Y, Lazurashvili N, Schmidt R, Hardtke HJ, et al. (2011) A segmenta-tion method to obtain a complete geometry model of the hearing organ. Hear Res 282: 25–34. doi: 10.1016/j.heares.2011.06.009 PMID: 21782915
20. Poznyakovskiy AA, Zahnert T, Kalaidzidis Y, Schmidt R, Fischer B, Baumgart J, et al. (2008) The creation of geometric three-dimensional models of the inner ear based on micro computer tomography data. Hear Res 243: 95–104. doi: 10.1016/j.heares.2008.06.008 PMID: 18625296
21. Nordenberg M, Sundberg J (2004) Effect on LTAS of vocal loudness variation. Logoped Phoniatr Vocol 29: 183–191. PMID: 15764212
22. Hammarberg B, Fritzell B, Gauffin J, Sundberg J, Wedin L (1980) Perceptual and acoustic correlates of abnormal voice qualities. Acta Otolaryngol 90: 441–451. PMID: 7211336
23. Omori K, Kacker A, Carroll LM, Riley WD, Blaugrund SM (1996) Singing power ratio: quantitative evaluation of singing voice quality. J Voice 10: 228–235. PMID: 8865093
24. PRAAT software. Available: http://www.fon.hum.uva.nl/praat/. Accessed 11 March 2015.
25. Kitamura T, Takemoto H, Honda K, Shimada Y, Fujimoto I, Syakudo Y, et al. (2005) Difference in vocal tract shape between upright and supine postures: Observations by an open-type MRI scanner. Acoust Sci Tech 26: 465–468.
26. Traser L, Burdumy M, Richter B, Vicari M, Echternach M (2014) Weight-bearing MR imaging as an option in the study of gravitational effects on the vocal tract of untrained subjects in singing phonation. PLoS One 9: e112405. doi: 10.1371/journal.pone.0112405 PMID: 25379885
27. Traser L, Burdumy M, Richter B, Vicari M, Echternach M (2013) The effect of supine and upright position on vocal tract configurations during singing{a comparative study in professional tenors. J Voice 27: 141–148. doi: 10.1016/j.jvoice.2012.11.002 PMID: 23380394
28. Roers F, Mürbe D, Sundberg J (2009) Predicted singers' vocal fold lengths and voice classification-a study of x-ray morphological measures. J Voice 23: 408–413. doi: 10.1016/j.jvoice.2007.12.003 PMID: 18395418
29. Vennard W, editor (1967) Singing: The Mechanism and the Technic.
30. Shipp T, Izdebski K (1975) Letter: Vocal frequency and vertical larynx positioning by singers and non-singers. J Acoust Soc Am 58: 1104–1106. PMID: 1194561

31. Detweiler RF (1994) An investigation of the laryngeal system as the resonance source of the singer's formant. J Voice 8: 303–313. PMID: 7858665

32. Andrade PA (2012) Analysis of male singers laryngeal vertical displacement during the first passaggio and its implications on the vocal folds vibratory pattern. J Voice 26: 665 e619-e624.

33. Story BH, Titze IR, Hoffman EA (2001) The relationship of vocal tract shape to three voice qualities. J Acoust Soc Am 109: 1651–1667. PMID: 11325134

34. Fleischer M, Pinkert S, Mattheus W, Mainka A, Murbe D (2014) Formant frequencies and bandwidths of the vocal tract transfer function are affected by the mechanical impedance of the vocal tract wall. Biomech Model Mechanobiol. 2014 Nov 23 [Epub ahead of print]
